# Rates and determinants of incident lacunes: A systematic review and meta-analysis

**DOI:** 10.1177/17474930261421046

**Published:** 2026-01-28

**Authors:** Fei Han, Ding-Ding Zhang, Joanna M Wardlaw

**Affiliations:** 1 Department of Neurology, Peking Union Medical College Hospital, Chinese Academy of Medical Sciences and Peking Union Medical College, Beijing, China; 2 Center for Prevention and Early Intervention, National Infrastructures for Translational Medicine, Institute of Clinical Medicine, Peking Union Medical College Hospital, Chinese Academy of Medical Science and Peking Union Medical College, Beijing, China; 3 Centre for Clinical Brain Sciences, Department of Neuroimaging Sciences, University of Edinburgh, Edinburgh, UK; 4 UK Dementia Research Institute at the University of Edinburgh, Edinburgh Medical School, Edinburgh, UK

**Keywords:** Cerebral small-vessel disease, lacune, incidence, risk factor, systematic review, meta-analysis

## Abstract

**Background:**

Lacunes of presumed vascular origin are a key imaging marker of cerebral small-vessel disease (cSVD), predicting stroke and dementia risk. Their incidence and determinants have not been systematically quantified across different populations, and implications for clinical research remain unclear.

**Aims:**

This study aims to estimate the annualized incidence of new lacunes across diverse populations, identify study-level factors contributing to heterogeneity, summarize patient-level risk factors for incident lacunes, and provide empirical data to inform sample size estimation for studies using incident lacunes as an imaging outcome.

**Summary of review:**

Thirty-one studies comprising 12,646 participants and 56,073 person-years were included. The pooled overall incidence was 3.27 per 100 person-years (95% CI, 2.12–4.42), ranging from 1.50 to 8.03 across populations. Rates were highest in cSVD patients (8.03; 95% CI, 3.8–12.27), intermediate in stroke and memory-clinic patients, and lower in community-based, hypertensive, and non-specific artery disease cohorts. Meta-regression showed that baseline lacune prevalence was positively associated with incidence (β = 0.057; 95% CI, 0.006–0.108; *P* = 0.031). At the individual level, male sex, baseline lacunes, hypertension, and diabetes were associated with higher risk. In cSVD populations, detecting a 30% relative risk reduction required 563, 867, and 1782 participants per arm for 3-, 2-, and 1-year follow-up, respectively.

**Conclusion:**

Incident lacune rates vary substantially across populations and are strongly influenced by baseline lacune burden and vascular risk factors. These findings provide context for population selection and sample size considerations in studies using incident lacunes as an imaging outcome.

## Graphical abstract

**Graphical abstract:**
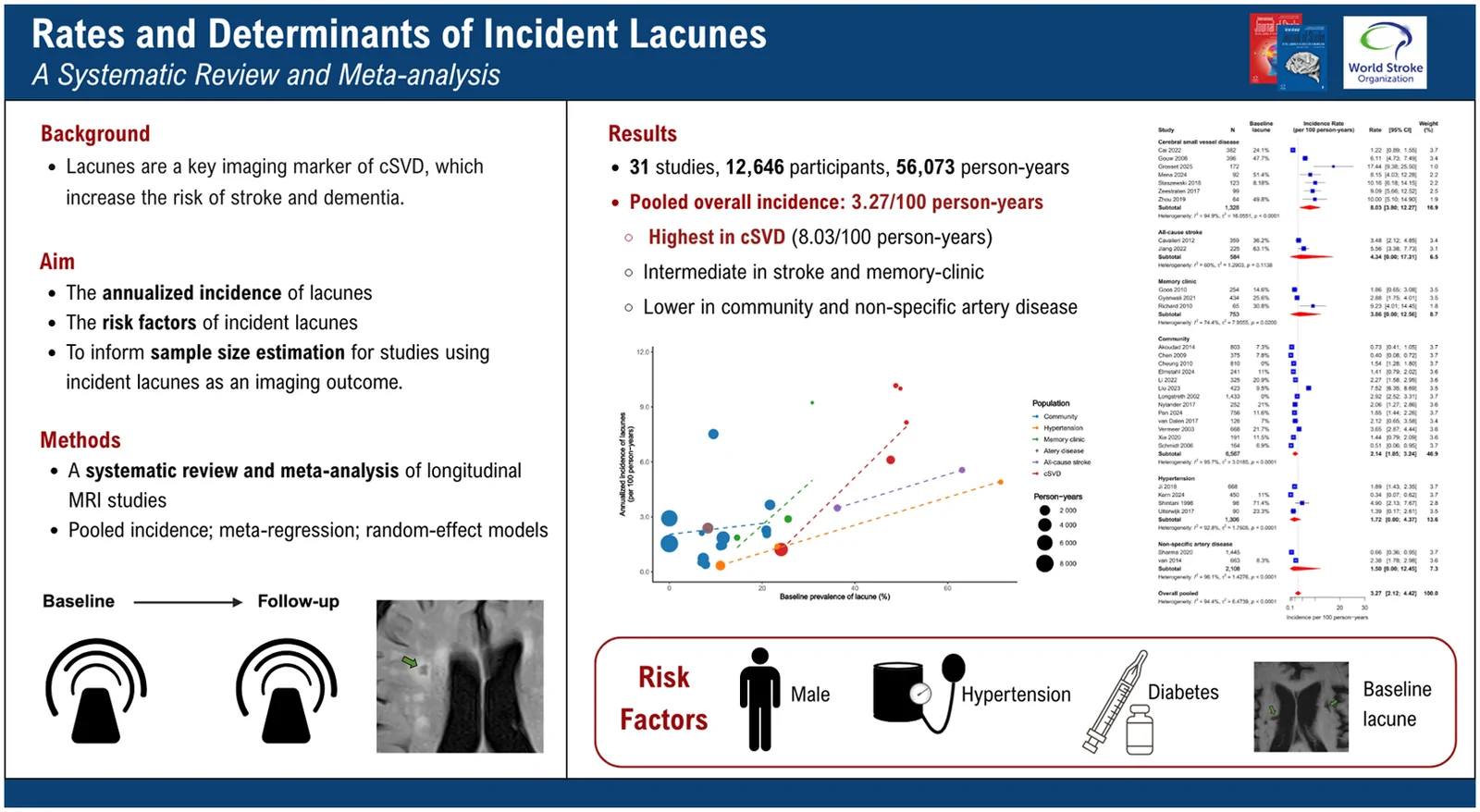

## Introduction

Cerebral small-vessel disease (cSVD) is a common cause of stroke and an important contributor to cognitive decline and vascular dementia.^1–3^ Lacunes of presumed vascular origin represent a key imaging marker of cSVD.^4^ Silent brain infarcts (SBI), mostly lacunes, increase the risk of subsequent stroke twofold to fourfold and double the risk of dementia, independent of cardiovascular risk factors.^5–7^ These clinically unrecognized but radiologically detectable lesions occur far more frequently than symptomatic strokes, with an incidence approximately fivefold higher in the general population and even greater among patients with prior stroke.^8,9^

Incident lacunes provide an objective MRI-based measure of structural brain injury progression and represent a pragmatic imaging outcome for longitudinal studies of cSVD. As visually defined lesions identifiable on MRI, they may help inform the evaluation of interventions in early-phase clinical trials, including both secondary prevention in patients with established cSVD and primary prevention in community populations.^10^

Despite their clinical and research significance, the incidence of lacunes and their determinants have not been systematically quantified. To address this gap, we conducted a systematic review and meta-analysis of longitudinal MRI studies to: (1) estimate annualized incidence rates of new lacunes across diverse populations; (2) identify study-level factors underlying heterogeneity; (3) summarize reported patient-level risk factors for incident lacunes; and (4) provide empirical sample size estimates for studies using incident lacunes as an imaging outcome.

## Methods

This systematic review followed the Preferred Reporting Items for Systematic Reviews and Meta-Analyses (PRISMA) guidelines^11^ and was registered with PROSPERO, the International Prospective Register of Systematic Reviews (CRD420251158772). The data that support the findings of this study are available from the authors upon reasonable request.

## Literature search and study eligibility

We systematically searched MEDLINE, Embase, and the Cochrane Library from inception to 25 September 2025, for full-text English-language articles. The search strategy combined terms related to incident lacunes, longitudinal follow-up, and brain MRI (Table S1). Reference lists of all eligible studies and relevant reviews were manually screened to identify additional records. Two reviewers (F.H. and D.Z.) independently screened all records for eligibility; any discrepancies were resolved through discussion and consensus.

We included longitudinal studies in adults, encompassing observational cohorts and randomized controlled trials (RCTs), that reported new (incident) lacunes on follow-up MRI, defined as subcortical, fluid-filled (cerebrospinal fluid-like) cavities <20 mm in diameter. Studies reporting only incident ischemic lesions (e.g. DWI-positive lesions or new non-cavitated white matter hyperintensities [WMH]) or only SBIs without separately defining lacunes were excluded. We also excluded those focusing on hereditary cSVD, cerebral amyloid angiopathy, or cohorts primarily investigating cardioembolic sources (e.g. atrial fibrillation, patent foramen ovale, valvular disease, and heart failure), cranial artery stenosis, or recent endovascular or cardiac procedures. Finally, studies with follow-up <6 months, sample size <50, as well as reviews, case reports, conference abstracts, and cross-sectional designs, were excluded.

## Data extraction

Data were extracted independently by two reviewers (F.H. and D.Z.) using a standardized template. When multiple publications described the same cohort, the article with the largest sample size was selected. Studies were categorized by population type, including community-based cohorts; cSVD cohorts, defined by clinical lacunar stroke, recent small subcortical infarcts (RSSI) on brain imaging, or chronic imaging markers (e.g. WMHs), with heterogeneous inclusion criteria; all-cause stroke populations; memory-clinic patients or individuals with cognitive complaints; participants with vascular risk factors such as hypertension; and those with non-specific artery disease. For each study, we recorded the baseline prevalence of lacunes and the number of MRI scans during follow-up. In RCTs, data from different trial arms were combined when no significant between-group differences were reported. Reported patient-level risk factors were extracted. Post-treatment indicators, such as achieved blood pressure or lipid levels, were also collected when reported.

## Quality assessment

Risk of bias was independently assessed by two reviewers (F.H. and D.Z.) using the modified Newcastle-Ottawa Scale.^12^ The scale comprises three domains: selection, comparability, and outcome, with a maximum score of 8 points.

## Statistical analysis

For each study, the annualized incidence of lacunes was calculated as the number of new lacunes divided by total person-years. Pooled incidence rates with 95% confidence intervals (CIs) were estimated using random-effects meta-analysis with each study weighted according to its sample size and accumulated person-years, using restricted maximum likelihood (REML) variance estimation and Hartung–Knapp adjustment. Prespecified subgroup analyses were conducted by study population, and heterogeneity quantified using I2 and τ2.

To explore sources of heterogeneity, multivariate mixed-effects meta-regression models were fitted with hypothesis-driven predictors, including study population, mean age, sex proportion, mean follow-up duration, and number of MRI scans. REML estimation with Knapp–Hartung adjustment was applied, and the proportion of explained variance was reported. The association between baseline prevalence and incidence of lacunes was evaluated using weighted linear regression. Study-level associations of imaging and study design factors (number of MRI scans, MRI field strength, and publication year) with incidence were further examined using Pearson correlation. For patient-level risk factors reported in ⩾2 studies, odds ratios (ORs) were pooled using random-effects models and presented as forest plots.

To informing sample size estimation, we modeled a hypothetical two-arm trial with balanced allocation, using incident lacunes as an imaging outcome. Based on pooled annual incidence rates, we estimated per-arm sample sizes required to detect relative risk reductions of 30%, 25%, 20%, 15% and 10% at 1-, 2-, and 3-year follow-up periods, assuming 80% power and two-sided α = 0.05. Estimates were further stratified by baseline lacune prevalence.

Publication bias was assessed using funnel plots and Egger’s test when more than 10 studies were available. All analyses were performed in R (version 4·4·2), with two-sided *p* < 0.05 considered significant.

## Results

Figure 1 shows the study selection process. Of 8406 records identified, 1960 duplicates were removed, leaving 6446 for title and abstract screening, of which 6304 were excluded. Among 142 articles reviewed in full text, 92 were excluded for being cross-sectional, focusing on large-artery or cardioembolic etiologies, or representing duplicate cohorts. Of 50 studies reporting incident infarcts, 19 were further excluded due to follow-up <6 months, sample size <50, or reporting only silent or covert infarcts rather than lacunes. Ultimately, 31 independent studies were included (Table 1), with detailed quality assessments provided in Tables S2 and S3 in the supplemental material.

**Figure 1.**
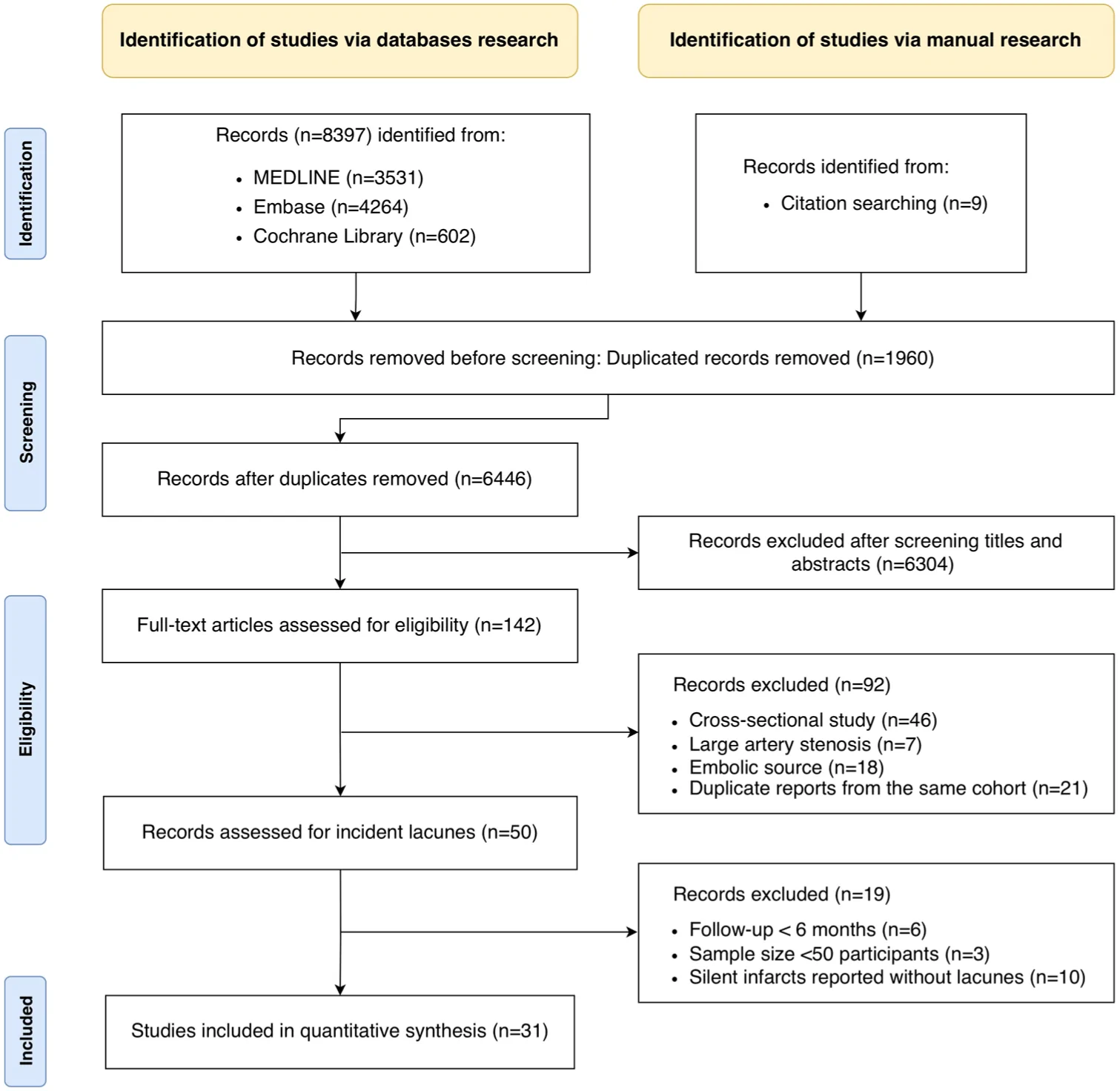
Preferred reporting items for systematic reviews and meta-analyses (PRISMA) flow diagram of study selection.

**Table 1.** Study characteristics.

Author, year	Country	Study name or site	N	Mean age	Men (%)	Mean follow-up (years)	New lacune (N)	Incidence of lacune/100 person-years	Baseline lacune (%)	No. of scans	Field strength	Slice thickness (T1WI, mm)	Size of lacune (mm)
**Community-based cohort**													
Akoudad, 2014^13^	Netherlands	Rotterdam study^*^	803	68.3	49	3.4	20	0.73	7.3	2	1.5 T	1.6	3–15
Chen, 2009^14^	Australia	PATH TLS	375	62.6	53	4	6	0.40	7.8	2	1.5 T	2	3–20
Cheung, 2010^15^	US	ARIC	810	61.6	40	10.5	131	1.54	0	2	1.5 T	5	3–20
Elmstahl, 2024^16^	Sweden	GAS	241	76.3	41	5.9	20	1.41	11	2	3 T	3–5	<10
Li, 2022^17^	Sweden	SNAC-K	325	70	40	5.7	42	2.27	20.9	3	1.5 T	1.5	3–15
Liu, 2023^18^	China	Chinese cohort	423	67	41	5	159	7.52	9.5	4	3 T	1	3–15
Longstreth, 2002^19^	US	CHS	1433	74	40	5	209	2.92	0	2	1.5 T	5	3–20
Nylander, 2017^20^	Sweden	PIVUS	252	75		5	26	2.06	21	2	1.5 T	3	3–15
Pan, 2024^21^	China	Shunyi study	756	53.7	35	5.6	78	1.85	11.6	2	3 T	1.3	3–15
Schmidt, 2006^22^	Austria	ASPS	164	63.9	46.3	6	5	0.50	6.9	3	1.5 T	5	<10
van Dalen, 2017^23^	Netherlands	preDIVA	126	77	47	3	8	2.12	7	2	3 T	1.2	3–15
Vermeer, 2003^24^	Netherlands	Rotterdam study^†^	668	71	48	3.4	83	3.65	21.7	2	1.5 T	5	3–20
Xia, 2020^25^	China	Chinese cohort	191	68	44	6.9	19	1.44	11.5	2	1.5/3 T	1.2	3–15
**Cerebral small-vessel disease**													
Cai, 2022^26^	Netherlands	RUN-DMC^‡^	382	64.1	57	11.2	52	1.22	24.1	4	1.5 T	5	3–15
Gouw, 2008^27^	Europe	LADIS^‡^	396	73.6	46	3.1	75	6.11	47.7	2	0.5/1.5 T	5	3–10
Grosset, 2025^28^	France	DHU-LAC^§^	172	63	72	0.6	18	17.44	/	2	3 T	/	3–15
Mena, 2024^29^	Spain	Moisès Broggi Hospital^§^	92	67	70	2	15	8.15	51.1	2	1.5 T	5	3–15
Staszewski, 2018^30^	Poland	SHEF-SVD^‡^	123	72.2	51	2	25	10.16	48.8	2	/	/	3–15
Zeestraten, 2017^8^	UK	SCANS^§^	99	68.9	67	3	27	9.09	/	4	1.5 T	/	3–15
Zhou, 2019^31^	China	CIRCLE^§^	64	56.3	52	2.5	16	10	49.8	2	3 T	1	3–15
**All-cause stroke**													
Cavalieri, 2012^32^	Austria	VITATOPS	359	64.3	64	2	25	3.48	36.2	2	1.5 T	/	⩽15
Jiang, 2022^33^	China	Ruijin Hospital	225	65.7	66	2	25	5.6	63.1	2	3 T	2	⩽20
**Memory-clinic patients**													
Goos, 2010^34^	Netherlands	VU University Medical Center, Amsterdam	254	66	52	1.9	9	1.86	14.6	2	1.0 T	5	3–15
Gyanwali, 2021^35^	Singapore	National University Hospital, Singapore	434	74	45	2	25	2.88	25.6	2	3 T	/	3–15
Richard, 2010^36^	Netherlands	EVA study	65	76	48	2	12	9.23	30.8	2	/	/	/
**Hypertension**													
Ji, 2018^37^	China	Shandong area	668	78	52	5.2	65	1.89	/	3	3 T	2	3–15
Kern, 2024^38^	US	SPRINT	450	67	63	3.9	6	0.34	11	2	3 T	/	3–15
Shintani, 1998^39^	Japan	Toride Kyodo General Hospital	98	64	50	2.5	12	4.9	71.4	4	0.5 T	/	/
Uiterwijk, 2017^40^	Netherlands	HYBRiD	90	56.2	57.8	4	5	1.39	23.3	2	1.5 T	/	3–15
**Non-specific artery disease**													
Sharma, 2020^41^	Global	COMPASS^||^	1445	71	77	2	19	0.66	/	2	1~3 T	3–5	⩽15
van, 2014^42^	Netherlands	SMART^#^	663	57	81	3.8	60	2.38	8.3	2	1.5 T	4	3–15

* Baseline scan: 2005–2006; ^†^Baseline scan: 1995–1996; ^‡^Chronic stage; ^§^Lacunar stroke or recent small subcortical infarct;^||^Stable coronary artery disease or peripheral artery disease; ^#^Coronary artery disease, cerebrovascular disease, peripheral artery disease, and/or an abdominal aortic aneurysm.

Abbreviations: cSVD, cerebral small-vessel disease; PATH TLS, PATH Through Life Study; ARIC, Atherosclerosis Risk in Communities Brain Magnetic Resonance Imaging Study; GAS, Good Aging in Skåne; SNAC-K, Swedish National study on Aging and Care in Kungsholmen; CHS, Cardiovascular Health Study; PIVUS, Prospective Investigation of the Vasculature in Uppsala Seniors; ASPS, Austrian stroke prevention study; preDIVA, Prevention of Dementia by Intensive Vascular Care; RUN-DMC, Radboud University Nijmegen Diffusion Tensor and Magnetic Resonance Cohort; LADIS, The Leukoaraiosis and Disability Study; SCANS, St George’s Cognition and Neuroimaging in Stroke; VITATOPS, The VITAmins TO Prevent Stroke; EVA, Evaluation of Vascular Care in Alzheimer’s Disease; SPRINT, Systolic Pressure Intervention Trial; HYBRiD, hypertension and brain damage study; COMPASS, Cardiovascular Outcomes for People Using Anticoagulation Strategies; SMART, Second Manifestations of ARTerial disease.

## Incidence of lacunes

Across 31 studies, a total of 12,646 participants were followed for 56,073 person-years, with a median follow-up of 3.4 years (range, 0.6–11.2). Publication bias was assessed within each population subgroup. Among community cohorts, no significant funnel-plot asymmetry was detected (Figure S1; Egger’s test: *t* = 2.12, *p* = 0.06).

The overall pooled annualized incidence was 3.27 (95% CI, 2.12–4.42) per 100 person-years. Incident lacune rates varied across populations (Figure 2 and Table 2). The highest was observed in cSVD cohorts (*n* = 1328; 8.03 per 100 person-years; 95% CI, 3.8–12.27), followed by all-cause stroke (*n* = 584; 4.34; 95% CI, 0–17.31) and memory-clinic patients (*n* = 753; 3.86; 95% CI, 0–12.56). Rates were lower in community cohorts (*n* = 6567; 2.14; 95% CI, 1.05–3.24), hypertensive patients (*n* = 1306; 1.72; 95% CI, 0–4.37), and in those with non-specific artery disease (*n* = 2108; 1.50; 95% CI, 0–12.45). In multivariate meta-regression adjusting for age, sex, number of MRI scans, and follow-up duration, cSVD cohorts showed significantly higher incidence rates than community-based and hypertension cohorts. Longer follow-up was associated with a lower annualized incidence (β = −0.19 per year; 95% CI, −0.30 to −0.07; *p* = 0.003), and the model explained 52.4% of between-study heterogeneity (F-statistic 4.10 on 9 and 20 degrees of freedom, *p* *=* 0.004).

**Figure 2.**
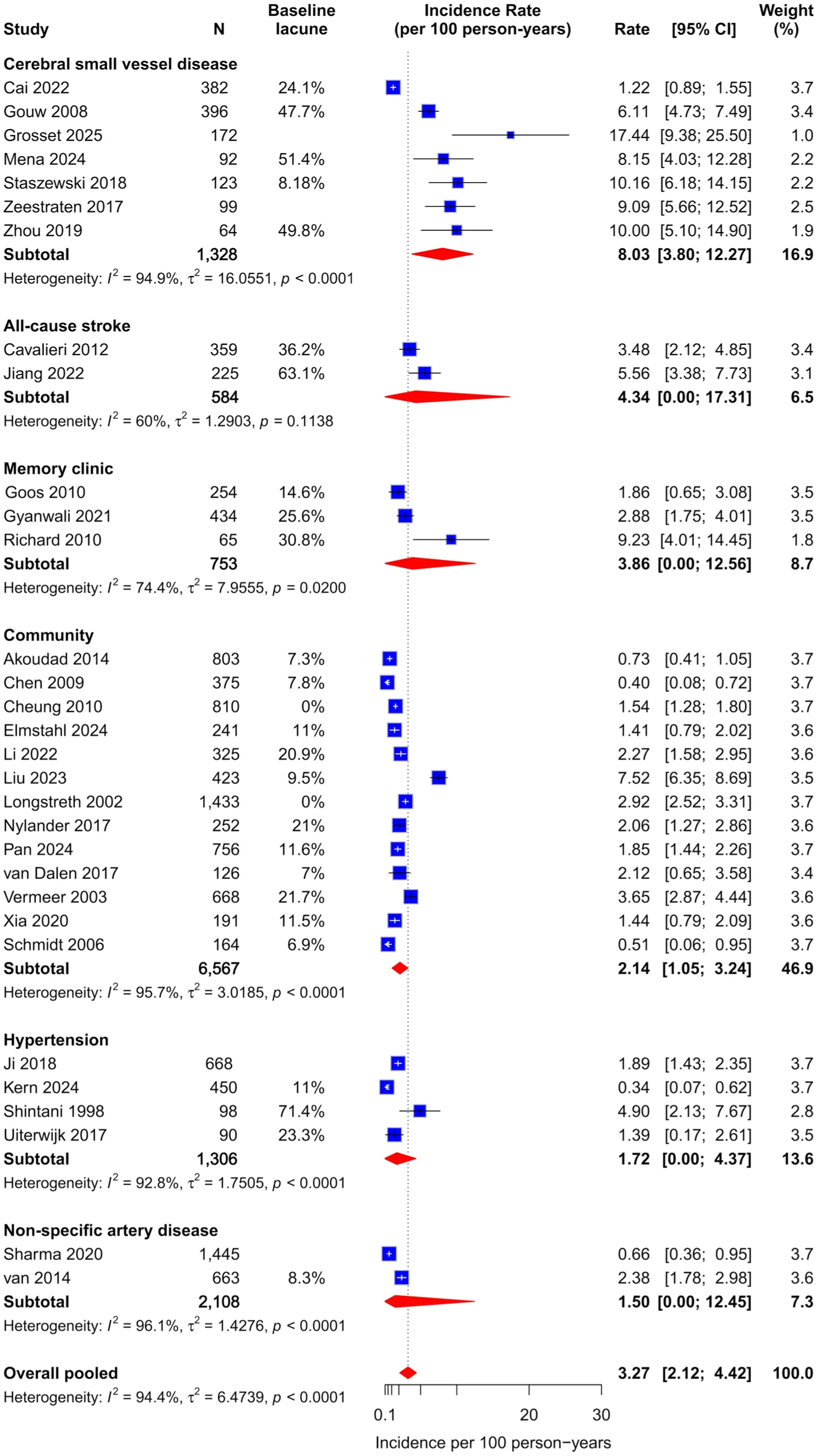
Pooled annualized incidence rates of lacunes by study population.

**Table 2.** Annualized lacune incidence for studies assessing different populations.

Population	No. of studies (patients)	Incident lacune /100 person-year	Versus community		Versus non-specific artery disease		Versus hypertension		Versus memory clinic		Versus stroke	
			RR (95% CI)^*^	*P* value	RR (95% CI)^*^	*P* value	RR (95% CI)^*^	*P* value	RR (95% CI)^*^	*P* value	RR (95 % CI)^*^	*P* value
cSVD	7 (1328)	8.03 (3.8–12.27)	2.97 (1.31–6.75)	0.012	2.42 (0.73–8.08)	0.142	3.78 (1.72–8.31)	0.002	2.51 (0.94–6.71)	0.065	1.55 (0.58–4.10)	0.362
Stroke	2 (584)	4.34 (0.00–17.31)	1.92 (0.58–6.36)	0.268	1.57 (0.43–5.74)	0.480	2.45 (0.79–7.54)	0.113	1.62 (0.46–5.75)	0.433		
Memory clinic	3 (753)	3.86 (0.00–12.56)	1.18 (0.49–2.83)	0.692	0.96 (0.19–4.89)	0.963	1.51 (0.53–4.30)	0.426				
Hypertension	4 (1306)	1.72 (0.00–4.37)	0.79 (0.33–1.88)	0.571	0.64 (0.16-2.57)	0.512						
Non-specific artery disease	2 (2108)	1.50 (0.00–12.45)	1.23 (0.25–5.99)	0.790								
Community	13 (6567)	2.14 (1.05–3.24)										

* Derived from multivariate mixed-effects meta-regression models adjusted for mean age, sex proportion, number of MRI scans, and mean follow-up duration. Abbreviations: cSVD, cerebral small-vessel disease; RR, relative risk; CI, confidence interval.

Baseline lacune prevalence was significantly associated with higher incidence in weighted regression (β = 0.057; 95% CI, 0.006–0.108; *p* = 0.031; adjusted *R*²= 0.14) and showed a similar positive relationship in Pearson correlation analysis (*r* = 0.67; 95% CI, 0.38–0.83; *p* < 0.001; Figure 3). In contrast, incidence was not significantly related to the number of MRI scans, MRI field strength, or publication year.

**Figure 3.**
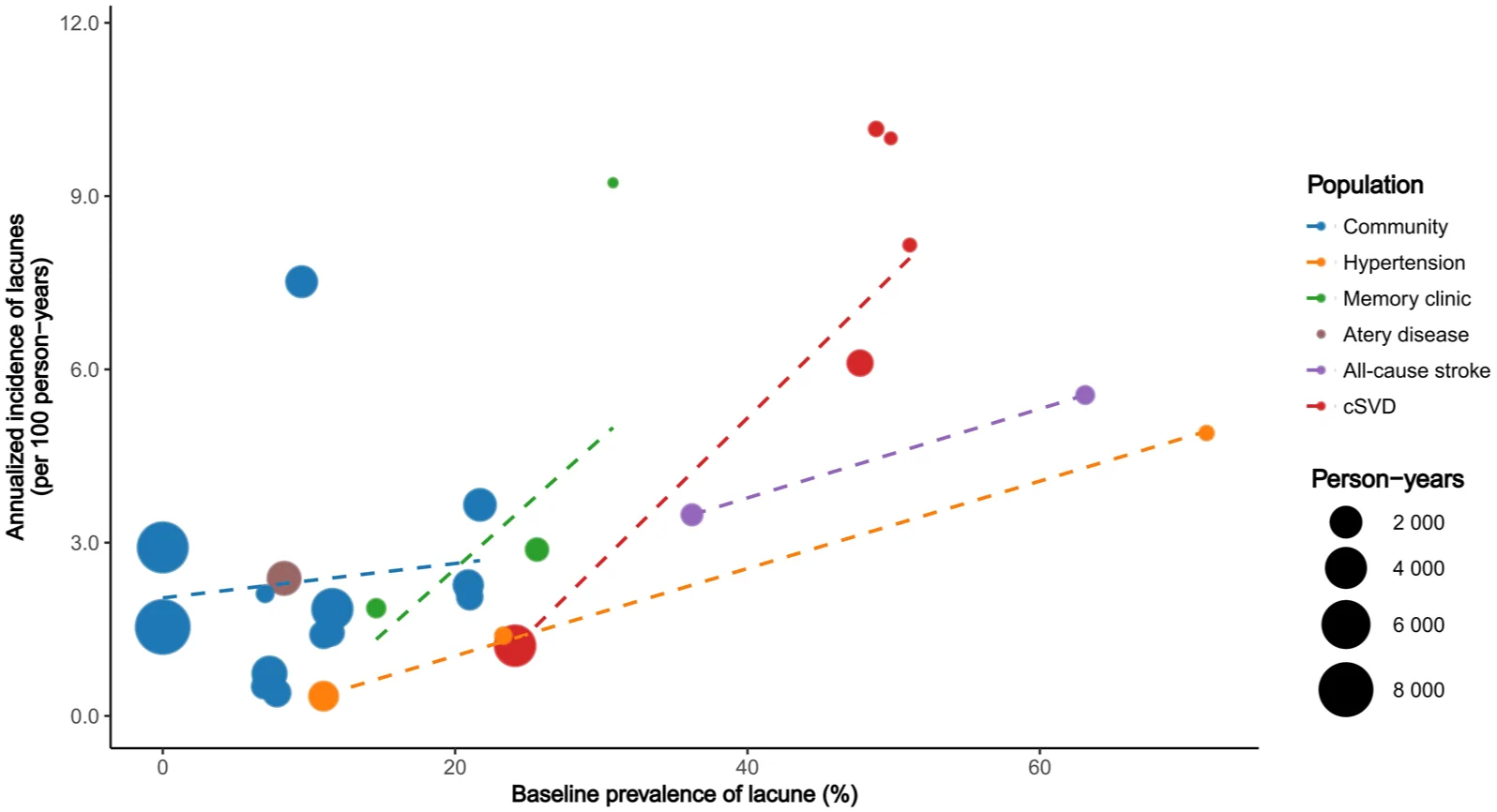
Bubble plots of annualized incidence of lacunes versus baseline lacune prevalence. Plot points are scaled by total person-years of follow-up and colored by study population. Dashed lines show fitted associations within each population group. Abbreviation: cSVD, cerebral small-vessel disease.

## Patient risk factors associated with incident lacunes

Across studies, baseline lacunes (OR, 2.24; 95% CI, 1.01–4.99) and male sex (OR, 1.43; 95% CI, 1.05–1.95) were significantly associated with an increased risk of incident lacunes, whereas age showed no significant effect (Figure 4(a)). Among modifiable factors (Figure 4(b)), hypertension (OR, 1.78; 95% CI, 1.24–2.56) and diabetes (OR, 1.69; 95% CI, 1.09–2.63) were associated with higher risk, while hyperlipidemia, smoking, and body mass index were not. Most studies did not account for how well these risk factors were controlled. In those that reported post-treatment indicators, higher blood pressure (OR, 1.30; 95% CI, 1.10–1.53) and elevated homocysteine levels (OR, 1.37; 95% CI, 1.00–1.87) were significantly associated with incident lacunes, whereas total cholesterol was not.

**Figure 4.**
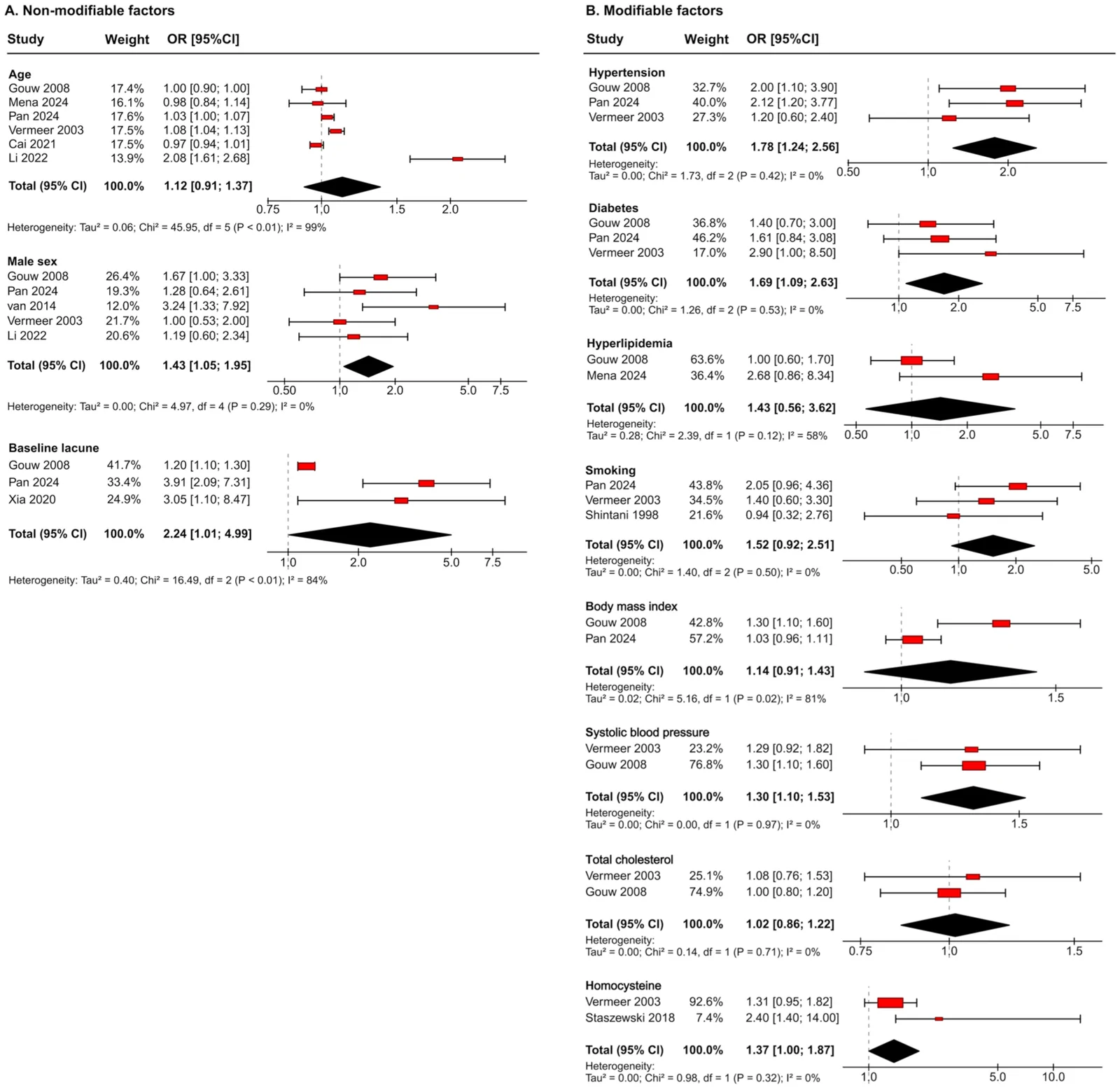
Effect sizes and meta-analysis results for risk factors of incident lacune: (a) non-modifiable factors and (b) modifiable factors. Abbreviations: OR, odd ratio; CI, confidence interval.

## Estimated sample sizes using incident lacunes as an imaging outcome

Sample size requirements varied markedly across populations (Table 3). Among patients with cSVD, detecting a 30% relative risk reduction in incident lacunes would require approximately 563, 867, and 1782 participants per arm for 3-, 2-, and 1-year follow-up, respectively. Stratification by baseline lacune prevalence revealed substantial variation (Figure S2): populations with a baseline prevalence of 45%–60% required approximately 75% fewer participants than those with a prevalence below 15% to detect the same 30% effect over 3 years (652 vs. 2716 per arm).

**Table 3.** Estimated prospective sample sizes (per arm) necessary to detect significant reductions in incident lacunes at different follow-up duration, stratified by study population, at power = 0.8 and α = 0.05.

Population	Follow-up time	Treatment effect sizes				
		30%	25%	20%	15%	10%
**Cerebral small-vessel disease**	3 years	563	831	1329	2415	5546
	2 years	867	1283	2057	3748	8630
	1 year	1782	2643	4247	7755	17,899
**All-cause stroke**	3 years	1081	1601	2568	4683	10,792
	2 years	1645	2440	3919	7155	16,510
	1 year	3339	4958	7975	14,575	33,673
**Hypertension**	3 years	2801	4157	6686	12,216	28,218
	2 years	4226	6276	10,097	18,458	42,656
	1 year	8502	12,631	20,332	37,187	85,975

## Discussion

This systematic review provides a comprehensive synthesis of the incidence and determinants of MRI-defined incident lacunes. Across 31 longitudinal studies involving more than 12,000 participants, the pooled overall incidence was 3.27 per 100 person-years, ranging from 1.5 to 8 across populations. The highest rates were observed in patients with cSVD, approximately 3.75 times higher than in older community-dwelling populations. Male sex, baseline lacune burden, and vascular risk factors, particularly hypertension and diabetes, were associated with increased risk. Furthermore, our analyses provide empirical data to inform the sample size estimation and the design of clinical studies that consider incident lacunes as an imaging outcome thereby offering methodological insights relevant to cSVD research.

Incidence varied substantially across populations. Patients with established cSVD, defined by clinical lacunar stroke or RSSI on imaging, or by chronic MRI markers, had the highest rates. Patients with all-cause stroke and those with cognitive complaints showed intermediate rates, whereas incidence was relatively low in community populations and hypertension cohorts. The non-specific artery disease group, which specifically included individuals with large-artery atherosclerosis (coronary artery and peripheral artery), showed the lowest incidence, indicating that large-vessel atherosclerosis does not necessarily translate into a high risk of lacunes or cSVD progression,^41,42^ consistent with the non-atheromatous pathology of cSVD. These findings underscore the importance of population selection when interpreting incidence estimates or designing studies using incident lacunes as an outcome.

The inverse association between follow-up duration and annualized incidence likely reflects a “front-loaded” risk pattern in cSVD. Early after an index event, for example, a clinical lacunar stroke, new ischemic lesions may indicate an active disease phase, with risk declining as the condition stabilizes. This interpretation is supported by a longitudinal study of patients with mild stroke enriched for cSVD, showing that although incident DWI-positive infarcts occurred throughout the year, most incident infarcts, particularly small subcortical ones, occurred within the first 3 months after the index stroke, despite optimal guideline-based secondary prevention.^43^ Such early clustering of incident ischemia may explain the higher annualized rates observed in studies with shorter follow-up, whereas longer follow-up intervals diluted this early peak. Therefore, sample size estimations based on annualized incidence rates should be interpreted with caution.

Both the between-study meta-regression and pooled individual-level analyses demonstrated that a higher baseline lacune burden was significantly associated with an increased risk of incident lacunes. This suggests that pre-existing lacunes are not merely markers of past injury but indicate an ongoing process of small-vessel damage or a predisposition to tissue cavitation and new lacune formation. It underscores the prognostic value of baseline MRI markers for risk stratification: patients with existing lacunes represent a higher-risk subgroup in whom intensive vascular risk factor management and closer imaging surveillance may be particularly warranted.

These findings have implications for designing clinical studies and trials that consider incident lacunes as a pragmatic imaging outcome. Baseline lacune burden should be considered both as a potential effect modifier and as a key determinant of sample size. The disease stage at recruitment warrants careful consideration. Enrolling patients at an early, “community-population” stage may enable intervention before irreversible damage occurs, but the lower incidence necessitates larger sample sizes or longer follow-up to detect meaningful effects. Conversely, recruiting patients with a higher burden, for example, those with “active cSVD,” yields higher event rates and smaller sample requirements, but the therapeutic benefits may diminish once microvascular damage has advanced. Balancing early intervention against trial feasibility will, therefore, be critical. Moreover, combining incident lacunes with other imaging markers, such as WMH progression, or incorporating them into summary cSVD scores, may further improve research efficiency and clinical relevance.^44,45^

Several explanations may account for the lack of an independent association between age and incident lacunes. Many included studies enrolled relatively older participants with restricted age ranges, and age is closely correlated with baseline lacune burden, which may attenuate its independent effect in adjusted analyses. Survivor bias may also contribute, with individuals at higher risk being underrepresented at older ages. Furthermore, the type of participant recruitment setting could play a role: studies recruiting participants from stroke clinics, for example, might enroll individuals who are more likely to present with lacunes and develop incident lacunes at younger ages, thereby skewing the age-lacune association.

The association between male sex and incident lacunes aligns with prior evidence that men are more likely to develop lacunar stroke and exhibit a greater cSVD burden, consistent with meta-analytic data demonstrating a male predominance in cSVD severity.^46^ This sex-related susceptibility may reflect underlying biological differences, including lipid metabolism and endothelial function. Supporting this, a large metabolomic study identified sex-specific associations between circulating metabolites, specifically, higher levels of hydroxyphenylpyruvate and lower levels of lysophosphatidylcholines and hydroxysphingomyelins, that were strongly associated with WMH in men but not in women.^47^

Hypertension, diabetes, higher achieved blood pressure, and homocysteine level were associated with a higher risk of incident lacunes, highlighting the importance of vascular risk management in slowing cSVD progression and preventing recurrent injury. This aligns with findings from the Systolic Blood Pressure Intervention Trial, which reported less WMH progression with intensive blood pressure reduction.^48^ However, no RCTs have yet demonstrated that blood pressure lowering, smoking cessation, diabetes management, or lifestyle modification can effectively reduce the incidence of lacunes.^49^

This study has several notable strengths. We conducted a comprehensive, protocol-registered systematic review and meta-analysis in accordance with PRISMA guidelines. All records were independently screened by two reviewers, with discrepancies resolved through consensus. A rigorous risk-of-bias assessment was applied, and random-effects models with Knapp–Hartung adjustment were used to generate robust pooled estimates. Beyond study-level meta-regression, we synthesized individual-level risk factors, providing consistent evidence from both between-study and within-study analyses.

Several limitations should be acknowledged. First, heterogeneity in MRI protocols, omission of small lacunes, and differences in follow-up imaging frequency may have influenced pooled incidence estimates. Stratified analyses by ethnicity or lacune location were not feasible because of inconsistent reporting and limited sample sizes within subgroups. Furthermore, MRI-defined lacunes may arise from heterogeneous underlying processes and should, therefore, be interpreted as a pragmatic composite imaging outcome rather than a biologically homogeneous entity. Second, although annualized incidence rates facilitated comparison across studies, this approach assumes a constant rate over time and may not fully capture periods of hyperactive or quiescent disease activity. Third, data for several individual risk factors were limited. Finally, the work does not apply to incident DWI-positive lesions or small subcortical infarcts that did not cavitate. Moreover, some studies reported only SBIs or ischemic lesions without separately defining lacunes; excluding these studies may have introduced selection bias.

In conclusion, incident lacunes occur at markedly variable rates across populations and are closely associated with baseline lacune burden. Male sex, hypertension, and diabetes are key risk factors. These findings highlight the importance of controlling modifiable vascular risk factors and provide empirical data to inform sample size estimation and the design of studies using incident lacunes as an imaging outcome.

## Supplemental Material

sj-pdf-1-wso-10.1177_17474930261421046 – Supplemental material for Rates and determinants of incident lacunes: A systematic review and meta-analysis Supplemental material, sj-pdf-1-wso-10.1177_17474930261421046 for Rates and determinants of incident lacunes: A systematic review and meta-analysis by Fei Han, Ding-Ding Zhang and Joanna M Wardlaw in International Journal of Stroke

## Data Availability

The data will be made available upon reasonable request.
