# Supplementary material for: Rates and determinants of incident lacunes: A systematic review and meta-analysis
Source: Int J Stroke. 2026 Jan 28;21(9):1299–310. doi: 10.1177/17474930261421046 (PMC13672885; doi:10.1177/17474930261421046)

Supplementary Materials

Table S1. Search strategy
Table S2. Quality assessment of studies reporting incidence of lacunes
Table S3. Quality assessment of studies reporting risk factor of incident lacunes
Figure S1. Funnel plots of the 13 community-based studies for publication bias assessment.
Figure S2. Heatmap of estimated prospective sample sizes necessary to detect significant reductions in incident lacunes, stratified baseline lacune prevalence.

Table S1. Search strategy

**MEDLINE (PubMed)**
("incident" OR "incidental" OR "incidence" OR "occurrence" OR "new" OR "recurren*") AND ("infarct*" OR "microinfarct*" OR "lacun*" OR "Ischemic lesion*" OR "Ischemia" OR "Ischaemic lesion*" OR "Ischaemia") AND ("longitudinal" OR "serial" OR "follow-up" OR "follow up" OR "baseline" OR "month*" OR "1 year" OR " 2 year*" OR "3 year*" OR "4 year*" OR "5 year*" OR "10 year*") AND ("MRI" OR "magnetic resonance") AND ("brain" OR "cerebral")
**Embase**
(incident OR incidental OR incidence OR occurrence OR new OR recurren*)<br>AND (infarct* OR microinfarct* OR lacun* OR "Ischemic lesion*" OR Ischemia OR "Ischaemic lesion*" OR Ischaemia)<br>AND (longitudinal OR serial OR "follow-up" OR "follow up" OR baseline OR month* OR "1 year" OR "2 year*" OR "3 year*" OR "4 year*" OR "5 year*" OR "10 year*")<br>AND (MRI OR "magnetic resonance")<br>AND (brain OR cerebral)
**Cochrane library**
(incident OR incidental OR incidence OR occurrence OR new OR recurren*)<br>AND (infarct* OR microinfarct* OR lacun* OR "Ischemic lesion*" OR Ischemia OR "Ischaemic lesion*" OR Ischaemia)<br>AND (longitudinal OR serial OR "follow-up" OR "follow up" OR baseline OR month* OR "1 year" OR "2 year*" OR "3 year*" OR "4 year*" OR "5 year*" OR "10 year*")<br>AND (MRI OR "magnetic resonance")<br>AND (brain OR cerebral)

Table S2. Quality assessment of studies reporting incidence of lacunes

Study	Selection			Comparability	Outcome			Total score	Quality
	Sampling	Exposure ascertainment	Baseline screening	Adjustment	Outcome assessment	Length of follow-up	Adequacy of follow-up[a]		
Akoudad, 2014	1	1	1	NA	1	1	1	6	Good
Cai, 2022	1	1	1	NA	1	1	1	6	Good
Cavalieri, 2012	1	1	1	NA	1	1	1	6	Good
Chen, 2009	1	1	1	NA	1	1	1	6	Good
Cheung, 2010	1	1	1	NA	1	1	0	5	Good
Elmstahl, 2024	1	1	1	NA	1	1	0	5	Good
Goos, 2010	0	1	1	NA	1	1	0	4	Good
Gouw, 2008	1	1	1	NA	1	1	0	5	Good
Grosset, 2025	1	1	1	NA	1	1	0	5	Good
Gyanwali, 2021	1	1	1	NA	1	1	1	6	Good
Ji, 2018	1	1	1	NA	1	1	1	6	Good
Jiang, 2022	1	1	1	NA	1	1	1	6	Good
Kern, 2024	1	1	1	NA	1	1	0	5	Good
Li, 2022	1	1	1	NA	1	1	0	5	Good
Liu, 2023	1	1	1	NA	1	1	1	6	Good
Longstreth, 2002	1	1	1	NA	1	1	0	5	Good
Mena, 2024	1	1	1	NA	1	1	1	6	Good

Nylander, 2017	1	1	1	NA	1	1	0	5	Good
Pan, 2024	1	1	1	NA	1	1	0	5	Good
Richard, 2010	1	1	1	NA	1	1	0	5	Good
Schmidt, 2006	1	1	1	NA	1	1	0	5	Good
Sharma, 2020	1	1	1	NA	1	1	1	6	Good
Shintani, 1998	0	1	1	NA	1	1	0	4	Good
Staszewski, 2018	1	1	1	NA	1	1	1	6	Good
Uiterwijk, 2017	1	1	1	NA	1	1	1	6	Good
van Dalen, 2017	1	1	1	NA	1	1	0	5	Good
van, 2014	1	1	1	NA	1	1	0	5	Good
Vermeer, 2003	1	1	1	NA	1	1	0	5	Good
Xia, 2020	1	1	1	NA	1	1	0	5	Good
Zeestraten, 2017	1	1	1	NA	1	1	1	6	Good
Zhou, 2019	1	1	1	NA	1	1	0	5	Good

[a] adequacy of follow-up: 1 point was awarded if at least 75% of the baseline participants completed follow-up.

Table S3. Quality assessment of studies reporting risk factor of incident lacunes

Study	Selection			Comparability	Outcome			Total score	Quality
	Sampling	Exposure ascertainment	Baseline screening	Adjustment	Outcome assessment	Length of follow-up	Adequacy of follow-up[a]		
Cai, 2022	1	1	1	1	1	1	1	7	Good
Gouw, 2008	1	1	1	2	1	1	0	7	Good
Li, 2022	1	1	1	1	1	1	0	6	Good
Mena, 2024	1	1	1	2	1	1	1	8	Good
Pan, 2024	1	1	1	2	1	1	0	7	Good
Vermeer, 2003	1	1	1	2	1	1	0	7	Good
Xia, 2020	1	1	1	2	1	1	0	7	Good

[a] adequacy of follow-up: 1 point was awarded if at least 75% of the baseline participants completed follow-up.

Figure S1. Funnel plots of the 13 community-based studies for publication bias assessment.

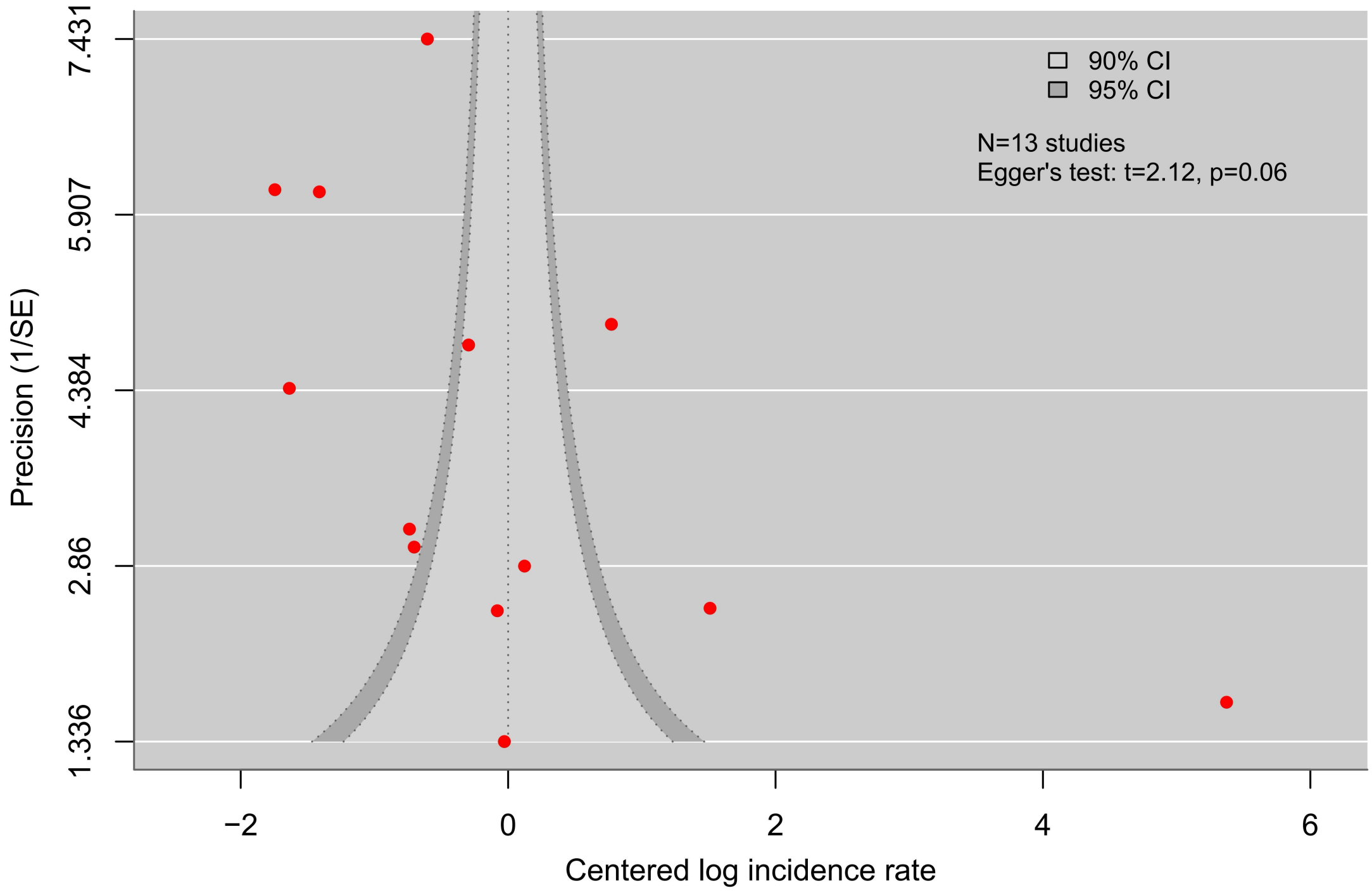

Figure S2. Heatmap of estimated prospective sample sizes necessary to detect significant reductions in incident lacunes, stratified baseline lacune prevalence. Numbers represent the required participants per arm; color intensity reflects sample size on a logarithmic scale

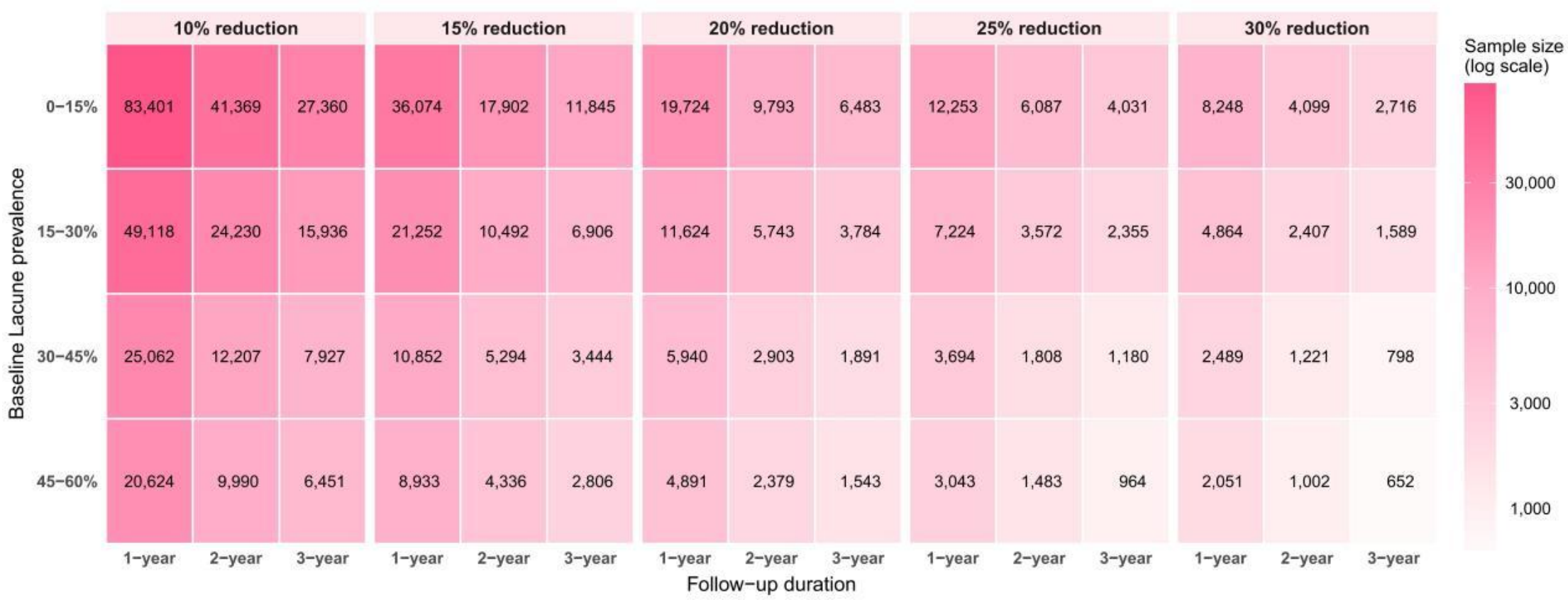

Supplement: Supplementary file 1 [file sj-pdf-1-wso-10.1177_17474930261421046.pdf]
